# National and International Meat Inspection1Read before the Twenty-seventh Annual Meeting of the United States Veterinary Medical Association, at Chicago, September 17th, 1890.

**Published:** 1890-11

**Authors:** Olof Schwartzkopff


### NATIONAL AND INTERNATIONAL MEAT INSPECTION.[1]

BY OLOF SCHWARTZKOPFF.

GENTLEMEN :—No occurrence in the history of the United States has had more significant relation to the veterinary profession of the country than the repeated endeavors of the legislatures of several States to make meat-inspection laws. Although these State laws have proved inefficient, and have not been sustained, it is not because the principle of such laws is wrong, but because the sanitary principles involved have not been sufficiently comprehended and guarded. Still, the movement in this direction has awakened great public interest, especially among agriculturists. In consequence, Congress has passed a bill providing for an inspection of meats for exportation into foreign countries. While this law is only one step in the right direction, it will, if carried out, benefit the stock-raising farmer of this country, and will also advance the cause of the veterinary profession of the United States, a feature which was least thought of by most of our national legislators.

Under these circumstances, then, it becomes us to use this occasion to discuss the principles which should govern sanitary meat-inspection laws both at home and abroad.

In dealing with the subject I will not enter into the history of meat-inspection, but only remark that it is not at all a modern idea, as stated by some newspapers and agricultural journals. In ancient times it was, of course, a religious rite, while it now

[1]Read before the Twenty-seventh Annual Meeting of the United States Veterinary Medical Association, at Chicago, September 17th, 1890.

belongs to sanitary science. Nor will I refer to the literature on the subject, which is quite comprehensive in some European languages. But I shall step at once into the proper theme and ask the following questions:

I. Is meat inspection necessary, and is it a sanitary measure?

II. What meat shall be regarded as wholesome for human food, and what as unwholesome?

III. How shall meat inspection be carried out?

I. The history of medicine tells us emphatically that there exists an intimate relation between our health and our use of animal food. It is an established physiological fact that an albuminous diet gives the human organism greater energy. While thus wholesome, meat may be regarded as of the greatest importance from the standpoint of national economy and also as one of those indirect civilizing powers, yet, meat from diseased animals brings forth many and dreadful dangers to society. Not only may we become temporarily sick by eating flesh of animals which were suffering, for instance, from a disease accompanied by high fever, and through the meat acquire those more or less injurious animal parasites such as tape worms and hydatides of the lungs and liver; but even life may be directly menaced with danger from the use of trichinous pork, or meat from tuberculous cattle or hogs, or from the most dangerous group of diseases in this direction, septicæmia and pyæmia.

But the general public can have no sufficient knowledge of these facts; it is impossible for the masses to acquire this particular knowledge in the other engagements of life. Individual self-protection seems impossible, consequently it falls upon the government, State or general, to provide a protection for our health and life alike in this direction, as is done in other matters of a sanitary nature. For this reason meat inspection becomes a sanitary precaution, demanding the attention of State and local boards of health.

II. In dealing with the question, "What meat shall be regarded as wholesome and what as unwholesome," I think it is essential that we should distinguish between inspection ante-mortem and post-mortem. I do not mean that we should separate these two inspections, for in all critical cases both are required for an intelligent decision. But in looking over the approved systems of veterinary police of the foremost European countries, we find that provision is made in most of them to directly forbid the consumption of meat of certain diseased animals. The reason for this action seems to be, first, that the use of meat in certain diseases is usually fatal; and, secondly, that if such meat be used the speedy extinction of certain infectious diseases is almost impossible.

Besides this, we know from statistics in meat inspection that in certain diseases the meat undergoes during life such alteration as to render it, without question, unfit for human food. In these cases we should not allow the regular slaughtering of the animals under any circumstances, but see that the carcasses are effectually destroyed. In other diseases the examination of the living animal, combined with post-mortem inspection, is necessary to enable us to properly decide whether the flesh may be used for human food or for industrial purposes only, or should be totally destroyed. Still, there remains a great variety of diseases in which the determination of the wholesomeness of the meat depends entirely upon an examination post-mortem. So large is the number of these diseases, that some veterinary officers of the great public slaughter-houses count them as being about 90 per cent. in the common routine of business.

With these points in view, I will undertake to make a classification of the diseases demanding special attention in the practice of meat inspection.

*I.—Diseases in which animals should be condemned, killed, and the carcasses effectually destroyed.*

(I.) Anthrax. (2.) Rabies. (3.) Septicæmia. (4.) Cattle plague. (5.) Glanders. (6.) Small-pox in sheep. (7.) Swine plague and hog cholera. (8.) Unborn animals.

*II.—Diseases in which slaughtering may be permitted to ascertain whether the whole or part of the meat is fit for human food or be used for industrial purposes, or be destroyed.*

(1.) Foot and mouth diseases. (2.) Tuberculosis in cattle, hogs and chickens. (3.) Actinomycosis in cattle. (4.) Icterus. (5.) Milk fever in cows. (6.) Hydrathorax and ascites. (7.) All diseases which are combined with high fever, general emaciation and debility; for instance, pneumonia, enteritis, uteritis, etc. (8.) Overheated and too young animals which should be kept for a further examination.

*III.—Diseases ascertainable only after slaughter, and in most cases by the use of the microscope.*

(1.) Parasitic diseases of meat: Cysticercus cellulosæ in hog and deer; cyst. c. tænia med. in cattle; trichinosis of hogs; actinomycosis of hogs; sporospermia and muscle distoma of hogs. (2.) Parasitic diseases of those organs which are used as human food: Brain, heart, lungs, liver, kidneys and intestines.

(*a.*) Brain: Coenurus cerebralis in cattle.

(*b.*) Heart: The different cysticerci.

(*c.*) Lungs: Strongylus micrurus in cattle; strongylus filaria in sheep; strongylus ovis-pulmonalis in sheep; strongylus paradoxus in swine; echinococcus.

(*d.*) Liver: Cysticercus tenuicollis; echinococcus; distoma hepaticum.

(*e.*) Kidneys.

(*f*) Intestines: Tapeworms and other intestinal worms that can be easily removed by turning the intestines and cleaning them with water.

This tabulation of diseases may serve for general purposes until through further scientific research certain diseases are better understood. Also, it is sufficient, as long as we limit ourselves strictly to the question whether meat is wholesome or unwholesome for human food. But it is sometimes asked if certain meat is of a proper nutritious quality? This raises another question, whether such jurisdiction belongs to veterinary science at all. It is probable that if meat inspection should become developed in this direction the chemist will be the proper man to decide. But if we pursue meat inspection within the limits of veterinary science, we are strictly in our line of work, and will come across very few difficulties. Still there occur cases which will not fit into the classification as suggested above, neither can any classification or direction as yet be made that will be a complete guide in all cases. From this reason alone, if not from others, it is obvious that only qualified veterinarians should be employed for this sanitary work; men who will decide such cases according to their knowledge of pathology, men to whose sphere of knowledge and judgment the whole question naturally belongs. And this will lead us to question

III. "How shall meat inspection be carried out?"

The practicability of a systematic inspection of meat and the efficient control of the meat supply requires:

(1.) Proper legislation to regulate the inspection and supply of meat. (2.) The erection of public abattoirs. (3.) Men of special scientific training to act as inspectors.

Without these provisions combined, any attempt at meat inspection will have very little value as a sanitary measure. We have witnessed the enactment of the so-called meat-inspection law in the States of Indiana, Minnesota, Colorado and the Territory of New Mexico. These laws were called meat-inspection laws, while they provided only for an inspection of the living animal in the stockyards, which consisted mainly in a superficial glance at the animals by the inspector from a distant point, condemning certain animals bearing conspicuous, but often harmless, blemishes, and overlooking others affected by dangerous maladies. On the whole, the performance amounted merely to a counting of the animals for the legal fee per head. I will read you an account of such inspection from a newspaper slip.

After discussing the enactment of the law, the reporter writes:

> "The inspector can inspect 1200 hogs and 600 cattle a day without trouble. How does he do it? The inspector, be it known, doesn't feel any pulse, look at any tongues, apply the stethoscope or go through any hoodoo incantations in determining the health of the steer, hog, sheep or calf. He just sizes the animal up, and if there is anything wrong he will see it instantly. In the case of cattle, which it is known are on the market for slaughter, he looks them over when they are weighed. It is also at the scales that all sheep and hogs are inspected. As they are driven on the scales they are counted, and again as the gates are opened and they troop off. The inspector stands up on the fence and if he sees a hog which does not wiggle about as though he felt tip-top, he is singled out and rejected. This is how animals, whose flesh is intended for human food, are inspected under the new law."

It does not need any comment on our part to show the absurdity of such inspection. But it works injury to the public, who believe that they are now protected from diseased meat and that no change of such law is needed. The butcher and stock dealer rather favor such inspection, as it does not cause them any loss or inconvenience. But we meet with the most vigorous opposition on the part of these very men when we de clare the necessity of inspecting the slaughter-house and the process of slaughtering and dressing. This they regard as an encroachment upon their private affairs. We may go all over the country and we will find that the entrance into the slaughter-house is not usually allowed, and we should not blame the butcher for this, for the slaughter-house is his sanctum sanctorum. Here he polishes his meat and makes the most of those manipulations which prepare the meat for the store. While it does not necessarily follow that anything wrong is done, and while I am personally convinced from years of official intercourse with butchers that in the main they are honest men, still there are some of them who are not so honest, and who will use without scruple diseased meat, even if they know it to be dangerous. In the slaughter-house, then, is just the place for the sanitarian to enter into a careful inspection; here he may often detect numerous pathological conditions which he never could have found in the living animal.

But even this is not the ultimatum of inspection; to accomplish our full purpose, we have to follow the meat into the store and direct such arrangements for its handling and storage as may be demanded by hygienic precaution, and especially in seasons favorable to the development of micro-organism. To do this, and to do it efficiently, we need such laws as will recognize and meet the emergencies just mentioned. While there must necessarily be left a certain liberty as to the decision of the sanitary officer, it is desirable that he have, as a basis for his action, well-defined law, which can be understood alike by him and the butcher and stock dealer. Such law is not only essential for the execution of meat inspection, but it will also save the butcher and the sanitary officer needless dispute and misunderstanding. The more elaborate such law is formulated the better it will work. It must define in unmistakable terms the duties of the sanitary officer and the butcher. And above all such law needs the advice of scientists as well as of the lawyer, It is one of our foremost duties to watch the development of these new laws, to instruct the public about the proper principles involved, and to use such professional influence upon legislators as to produce an efficient law. If this is done quietly but persistently, we cannot fail to produce much good.

The question for us to consider now is, where can meat inspection be carried out? Whoever has had the chance of visiting a slaughter-house, such as are found scattered all over the land, in city or country, must have felt disgusted at the prevalent condition of such places. Not to speak of the total absence of any hygienic arrangements, the unclean manner in which they are kept makes them a horror. Gentlemen, I feel sure that no meat inspection is possible nor advisable in such places; we cannot be expected to perform our duty in blood and dirt up to our ankles. If civilization should be extended anywhere, it is into the slaughter-house. But looking at this matter as leniently as possible, it remains a duty to condemn these places and to demand the erection of public abattoirs by the community. No expense is too large, no sacrifice too heavy to accomplish this end. The plan, location and erection of such public abattoirs should not be decided upon without the advice of a sanitarian, and an architect who can properly apply the principles of hygiene to such buildings.

As to the question, "who should be recognized as the proper expert in meat inspection?" there can be but one answer, namely, the veterinarian. To successfully perform both antemortem and post-mortem inspection requires a thorough knowl edgd of physiology and pathology of our domestic animals and their relation to the human race. As the physician is too much of a specialist to be compelled to pursue these studies, the other medical profession should consider them. This is specially proper since every educated veterinarian diligently follows the discoveries of medical science, whereas the physician is largely ignorant of what is going on in veterinary science.

To employ laymen as meat inspectors, such as stockmen and butchers, is a great mistake. I know from personal observation that such men will decide according to their feelings and prejudice rather than from knowledge. If asked why they condemn such and such meat, they will say, "I would not eat such flesh." The argument of some authorities on the employment of laymen is, that many veterinarians do not know more about it than these men. Now, that is erroneous. We may candidly admit that many veterinarians of this country are not up to a high standard of education; but however little they may have gained at college, they have gone through a systematic course which will at least enable them to know why they do so and why not otherwise; and, may I ask, is there any profession in the United States which has a uniform standard of education? Is there not the same difference of general and professional accomplishments in physicians, lawyers and ministers? Still, however great may be at present the perplexity about everything belonging to meat inspection in the heads of some of our legislators and their followers, we feel sure that the whole question will solve itself in time on its natural basis, and that the veterinarian of the United States will duly occupy the same position as a sanitarian that he effectually holds in European lands.

Having discussed the principles of meat inspection, it is not difficult to apply them to national or international legislation. In the United States there seems to be the tendency to deny the right of a single State to enact laws requiring meat inspection within its own limits, as this naturally restricts interstate commerce. It seems, then, as if the national government is the proper authority to carry out such laws. It does not matter much, from our professional standpoint, whether the national government or the States pass such laws. But if there be no uniform law otherwise, we should unite our influence in behalf of the interested public, to secure a national law which carries with it so much more authority and efficiency. It is to be deeply regretted that the bill recently introduced into the Senate by Senator Paddock, providing for national inspection of both cattle and hogs and their products intended for transportation from one State or Territory to another and for foreign exportation, has not been passed by Congress. This bill, with the exception of a very few points, was well adapted to serve the purpose, and would have been a judicious move. The failure of this bill to pass leaves us still without a national inspection law. Still, necessity will demand this or a similar measure in the near future.

There is an increasing disposition on the part of our legislators to promote the exportation of meat. That this can be accomplished only when we have a proper inspection throughout our country, and one which foreign nations can accept as adequate, is evident to anyone familiar with the strict meat-inspection laws of European countries.

Possibly, if meat inspection is brought into international negotiations, it may undergo some slight alterations, but its principles will stand, and we will have to adopt them.

The Edmunds Bill, which just passed Congress, provides for the inspection of salt pork for exportation. I give you Section 1 of this bill:

> "An Act providing for the inspection of meats for exportation, prohibiting the importation of adulterated articles of food and drink, and authorizing the President to make proclamation in certain cases, and for other purposes.
>
> "*Be it enacted by the Senate and House of Representatives of the United States of America in Congress assembled*, That the Secretary of Agriculture may cause to be made a careful inspection of salted pork and bacon intended for exportation, with a view of determining whether the same is wholesome, sound and fit for human food, whenever the laws, regulations or orders of the government of any foreign country to which such pork or bacon is to be exported shall require inspection thereof relating to the importation thereof into such country, and also whenever any buyer, seller or exporter of such meats intended for exportation shall request the inspection thereof."

This is the text referring to meat inspection, and then follow nine long sections prohibiting the importation of adulterated food and drink. While this may be evidently very necessary, it has no direct logical connection whatever with a meat-inspection law, and will look very suspicious in the eyes of the unprejudiced foreigner. But, to come back to the section read, is it not a mockery on what has just been stated at length? This alleged inspection of salt pork can only have reference to microscopical inspection, and you know with me that it is a most hopeless task to attempt such inspection. From the process of curing with salt the meat becomes so hard and rough that it is difficult, almost impossible, to prepare it for microscopical examination. Such methods are of very little value; indeed, for a careful microscopical examination, it is useless.

How then, can we expect, under such a law, that the foreign restrictions will be withdrawn? And if exportation should be attempted under such circumstances, what will be the result? Undoubtedly a second inspection on the part of foreign countries, which will add this expense to the price of the pork. The poor classes in several European countries are anxious to buy American pork, but will not be able to do so if the pork is not cheaper than that of the home country. This must reduce our export trade almost to nothing.

The European countries which import American pork are Germany, France, Norway and Sweden, Denmark and Italy. All these countries have at present restrictions against American pork. All these restrictions are based on sanitary grounds. Whether correctly or not I will not here undertake to discuss. Now, what change can we reasonably expect to result from the Edmunds law?

In closing my paper, I wish to appeal to your continued interest in the questions just discussed, and I especially appeal to the deans of the veterinary colleges of this country to provide for separate lectures on meat inspection, with practical exercises in public abattoirs; that if called upon we may have men able to undertake the responsibility that is connected with this sanitary measure. There can be no question that we shall witness some important legislation in regard to meat inspection in the coming years, and it should be our professional pride to intelligently advise our legislators as to the proper scientific standpoint of such laws.

As veterinarians, we are doubly interested in this question. It concerns us as members of the general public, but to a much greater degree does it interest us as members of the veterinary science, to which it gives great opportunities and adds great responsibilities.

